# Pentraxin 3 Is a Predictor for Fibrosis and Arterial Stiffness in Patients with Nonalcoholic Fatty Liver Disease

**DOI:** 10.1155/2016/1417962

**Published:** 2016-02-22

**Authors:** Kadir Ozturk, Omer Kurt, Tolga Dogan, Alptug Ozen, Hakan Demirci, Fatih Yesildal, Murat Kantarcioglu, Turker Turker, Ahmet Kerem Guler, Yıldırım Karslioglu, Battal Altun, Ahmet Uygun, Sait Bagci

**Affiliations:** ^1^Department of Gastroenterology, Gulhane School of Medicine, 06010 Ankara, Turkey; ^2^Department of Internal Medicine, Gulhane School of Medicine, 06010 Ankara, Turkey; ^3^Department of Radiology, Gulhane School of Medicine, 06010 Ankara, Turkey; ^4^Department of Medical Biochemistry, Gulhane School of Medicine, 06010 Ankara, Turkey; ^5^Department of Health Public and Epidemiology, Gulhane School of Medicine, 06010 Ankara, Turkey; ^6^Department of Pathology, Gulhane School of Medicine, 06010 Ankara, Turkey; ^7^Department of Internal Medicine, Kasımpasa Military Hospital, Istanbul, Turkey

## Abstract

*Objective*. The aim of the present study was to investigate whether pentraxin 3 (PTX3) can be a new noninvasive marker for prediction of liver fibrosis in patients with NAFLD. We also aimed to evaluate the relationship between PTX3 and atherosclerosis in patients with NAFLD.* Method*. Fifty-four male patients with biopsy-proven NAFLD and 20 apparently healthy male volunteers were included. PTX3 levels were determined, using an ELISA method (R&D Sysytems, Quantikine ELISA, USA). To detect the presence of subclinical atherosclerosis in NAFLD, measurements of CIMT, FMD, and cf-PWV levels were performed.* Results*. PTX3 levels in NAFLD patients with fibrosis were higher than both NAFLD patients without fibrosis and controls (*P* = 0.032 and *P* = 0.028, respectively), but there was no difference between controls and NAFLD patients without fibrosis in terms of PTX3 levels (*P* = 0.903). PTX3 levels were strongly correlated with cf-PWV (*r* = 0.359, *P* = 0.003), whereas no significant correlation was found with other atherosclerosis markers, CIMT and FMD.* Conclusion*. Elevated plasma PTX3 levels are associated with the presence of fibrosis in patients with NAFLD, independently of metabolic syndrome components. This study demonstrated that for the first time there is a close association between elevated PTX3 levels and increased arterial stiffness in patients with NAFLD.

## 1. Introduction 

The term nonalcoholic fatty liver disease (NAFLD) refers to the fatty infiltration of hepatocytes in the absence of significant alcohol intake [1]. Currently, a “two-hit hypothesis” has been proposed and recognized to explain the underlying mechanism in pathogenesis of NAFLD. A major mechanism is increased hepatic insulin resistance leading to hepatic steatosis. The subclinical inflammation process causes the steatosis to progress to hepatic inflammation and steatohepatitis [2]. Attention has been focused on distinguishing simple steatosis (SS) from nonalcoholic steatohepatitis (NASH) because NASH can lead to liver degeneration and finally progress to liver cirrhosis [3]. Currently, liver biopsy is accepted as the gold standard for both diagnosis of NAFLD and distinguishing NASH from SS. However, a noninvasive and clinical useful method is needed because of some limitations of liver biopsy such as being invasive, expensive, and needing hospitalization [4, 5]. Several clinical studies have focused on finding a biomarker which is most closely correlated with the severity of liver fibrosis in patients with NAFLD [6, 7] but already failed to prove a serum biomarker for accurately predicting the severity of liver fibrosis.

Recently, pentraxin 3 (PTX3), which is a member of the long pentraxin protein family, has been recognized as a new marker of localized vascular inflammation [8]. Unlike the C reactive protein (CRP), which is mainly produced in the liver, PTX3 is mainly produced in atherosclerotic vascular tissue [9]. Therefore, it is not surprising that PTX3 levels have been associated with unstable angina, myocardial infarction, heart failure, and cardiovascular mortality [10]. It is well known that NAFLD is an independent risk factor for cardiovascular disease (CVD) [11]. However, it has not yet been evaluated whether plasma PTX3 levels are associated with subclinical atherosclerosis in patients with NAFLD, so far.

The growing evidence suggests that CRP is a biomarker of low grade inflammatory process in patients with NASH [12]. However, data regarding the association between PTX3 and fibrosis in NAFLD is limited. Since the classic short pentraxin CRP is produced in the liver, it is thought that CRP levels in NAFLD may be a more sensitive marker for predicting severity of liver fibrosis when compared to PTX3 levels. Although three previous studies reported the relationship between plasma PTX3 concentrations and NAFLD, the results were conflicting and the value of plasma PTX3 for prediction of NAFLD was controversial [13–15].

The aim of the present study was to investigate whether PTX3 can be a new noninvasive marker for prediction of liver fibrosis in patients with NAFLD. We also aimed to evaluate the relationship between PTX3 and atherosclerosis in patients with NAFLD, by measuring carotid femoral pulse wave velocity (cf-PWV), carotid intima media thickness (CIMT), and flow mediated dilatation (FMD).

## 2. Materials and Methods

### 2.1. Study Population

In this cross-sectional study, fifty-four male patients with biopsy-proven NAFLD were included. Patients were eligible for inclusion if they had elevated liver enzymes for at least 6 months, ultrasonographically bright liver, and a histological diagnosis of fatty infiltration in liver biopsy. The patients were excluded if they were reported to consume more than 20 g of alcohol daily. Further, subjects with diabetes mellitus (DM), hypertension (HT), CVD, presence of other liver diseases (viral hepatitis, autoimmune hepatitis, drug-induced liver damage, etc.), inflammatory disorders (rheumatoid arthritis, systemic lupus erythematosus, etc.), infectious disease, and malignancy were excluded from the study. The control group consisted of 20 apparently healthy male volunteers who were matched to the patients with respect to age and gender.

Detailed information including current medications and smoking status was taken from each participant. The weight and height of the subjects were measured after the subjects had removed their shoes and any heavy clothing. Body mass index (BMI) was calculated as weight (kg) divided by height squared (m^2^). Waist circumference was measured at the midway point between the lowest rib margin and the iliac crest. Hip circumference was obtained at the widest point between hip and buttock. Blood pressure measurements were done with a sphygmomanometer with the patient in the seated position using the right arm after 5 min rest. The average of three measurements was accepted as systolic and diastolic pressures. HT was defined as either being treated with antihypertensive agents or having systolic and/or diastolic blood pressure of ≥140/90 mmHg.

Written informed consent was obtained from all participants before entering the study, and the study was approved by the Ethical Committee of Gulhane School of Medicine.

### 2.2. Biochemical Analyses

Blood sampling was done after 12 h fasting. Serum aspartate aminotransferase (AST), alanine aminotransferase (ALT), *γ*-glutamyltransferase (GGT), fasting plasma glucose (FPG), uric acid, HDL cholesterol (HDL-c), triglyceride (TG), and creatinine were determined by spectrophotometric methods with commercially available Olympus reagents (Beckman Coulter Inc., CA, USA) using an Olympus AU2700 (Beckman Coulter, USA) autoanalyzer. LDL cholesterol (LDL-c) was calculated by using Friedewald's equation, if TG level was lower than 400 mg/dL [16]. If not, LDL-c measurement procedure was employed. Serum high sensitivity-CRP levels (hs-CRP) were measured using immunoturbidimetric method.

Patients and healthy subjects underwent a standard 75 g oral glucose tolerance test (OGTT). Measurements of glucose were performed at 0 and 120 min. DM (fasting plasma glucose of ≥126 mg/dL or 2 h OGTT of ≥200 mg/dL) was classified according to American Diabetes Association guidelines [17]. The fasting insulin level was measured in duplicate by the chemiluminescence immunoassay method using reagents from Siemens Healthcare Diagnostics Inc. (NY, USA) on an Advia Centaur XP platform. Insulin resistance was estimated using a modified homeostasis model assessment of insulin resistance (HOMA-IR) index, calculated as follows: fasting plasma insulin (mU/mL) × fasting plasma glucose (mg/dL)/405 [18].

### 2.3. Pentraxin 3 Assay

Blood samples of patients with NAFLD were drawn at the same day of liver biopsy procedures. All venous samples were centrifuged at 2000 g for 10 min to obtain plasma samples. The plasma samples were aliquoted and stored at −80°C till the analysis. PTX3 levels were determined, using an ELISA method (R&D Systems, Quantikine ELISA, USA), by following the instructions in the manual, provided by the manufacturer. An ELISA plate reader (model ELx800 bioelisa microplate reader, Biokit, Spain) was used to measure the color intensity according to the instructions in manual provided by the manufacturer, and PTX3 level of each sample was determined.

### 2.4. Liver Histology

Liver biopsies were analyzed by a single expert pathologist blinded to the patients' clinical results. The samples were scored according to the categories established by Kleiner et al. [19]. Briefly, degree of steatosis was scored using the following scale: 0 (<5%), 1 (5–33%), 2 (33–66%), and 3 (>66%). Foci of lobular inflammation were defined as two or more inflammatory foci (averaged from 3-4 fields) and scored as: 0 (no foci), 1 (<2 foci), 2 (2–4 foci), and 3 (>4 foci). Hepatocyte ballooning was scored according to the number of ballooning cells: 0 (none), 1 (few), and 2 (many). Histopathological features were graded according to the NAFLD activity score (NAS), in which a score of ≥5 was defined as NASH. Stage of fibrosis was scored as follows: 0 (none), 1 (zone 3 perisinusoidal or portal fibrosis), 2 (zone 3 perisinusoidal and periportal fibrosis), 3 (bridging fibrosis), and 4 (cirrhosis).

### 2.5. Assessment of Arterial Stiffness

Carotid femoral pulse wave velocity (cf-PWV; an index of arterial stiffness) was measured in the supine position after resting for 5 minutes using an automatic waveform analyzer (TensioMed Ltd., Budapest, Hungary). A single experienced internist who was blinded to the clinical characteristics of the participants performed the measurements. Participants did not consume any food or drink and did not smoke for at least the 30 minutes leading up to the measurement period. The distance between the jugular notch and the symphysis pubis of each individual was measured, and the data were recorded on the device. During the measurement period, brachial artery occlusion was made, and the blood flow was ceased as a part of the process.

### 2.6. Assessment of Endothelial Dysfunction

Endothelial dysfunction (ED) was determined from endothelium-dependent vasodilatation of brachial artery, using high resolution ultrasound to measure FMD. Measurements were made by a single observer with an 18 MHz linear-array transducer on M mode (Siemens, Acuson S3000). A cuff was placed at the upper arm approximately 2–4 cm above the antecubital crease. The first baseline arterial diameter was measured. A pneumatic tourniquet was then inflated to 200 mmHg with obliteration of the radial pulse. After 3 min the cuff was deflated and the arterial diameter was measured. FMD at 1 min after ischemia [i.e., 100 × (Diameter (1 min) − Diameter(basal)/ Diameter(basal))] was used to represent spontaneous endothelial function.

### 2.7. Assessment of Carotid Intima Media Thickness

For the determination of intima media thickness (IMT), high resolution B mode ultrasonography (Siemens, Acuson S3000, Germany) with an 18 MHz linear-array transducer was used. The region of interest (ROI) for the measurement of IMT of the bilateral common carotid arteries (CCA) was selected 1 cm proximal to the CCA bifurcation. Longitudinal static images were analyzed using semi-automated software (Syngo Arterial Health Package). The transducer was manually placed on a 1 cm segment of the ROI and the IMT was then automatically measured by calculating the distance between the lumen-intima and the media-adventitia interfaces in the far wall of the ROI. The CIMT of both left and right CCA was measured only once, and the CIMT value was calculated by averaging measurements of the left and right common carotid IMT.

### 2.8. Statistical Analysis

SPSS (Statistical Package for the Social Sciences ver. 17.0, SPSS Inc., Chicago, IL, USA) computer program was used for all statistical calculations. Results were reported as the mean ± standard deviation (SD), frequency, and percentage. The Kolmogorov-Smirnov test was used to determine the distribution characteristics of continuous variables. The normally distributed variables were compared with one-way ANOVA for multiple-group comparison and a Tukey's post hoc test. The Kruskal-Wallis test was used for multiple-group comparison of variables without normal distribution, and a Bonferroni-adjusted Mann-Whitney *U* test was used for post hoc analysis. Categorical variables were compared by chi-square or Fisher's exact test. Student's *t*-test was used for binary comparisons of continuous variables. Pearson correlation analysis was used to evaluate the relationship between continuous variables. A multivariate analysis of variance was used to assess the effects of BMI and other clinical factors on PTX3. Statistical significance was defined as *P* < 0.05.

## 3. Results 

Demographic, biochemical, and vascular characteristics of 54 patients with biopsy-proven NAFLD and 20 healthy subjects were shown in Table 1. There was no significant difference in age, gender, and smoking status between the two groups. No significant difference also existed in levels of creatinine, HDL-c, TG, PTX3, and CIMT between the groups. Patients with NAFLD had higher BMI, WC, HC, SBP, DBP, AST, ALT, GGT, FPG, 2 h OGTT, uric acid, LDL-c, insulin, HOMA-IR, and hs-CRP compared to control subjects (*P* < 0.05). Additionally, patients with NAFLD were more likely to have endothelial dysfunction and increased arterial stiffness than healthy subjects (*P* = 0.014 and *P* = 0.006, resp.) (Table 1).

**Figure 1:**
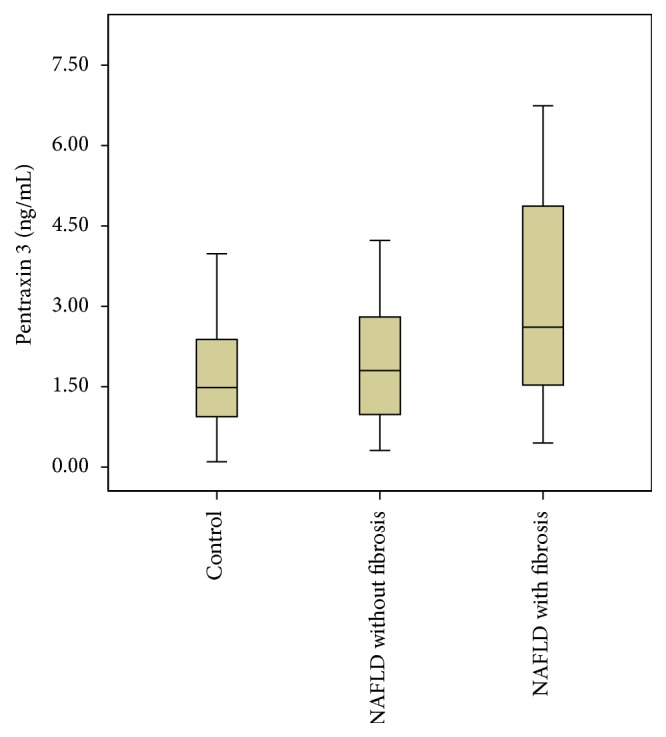
Pentraxin 3 concentrations (ng/mL) in the plasma of NAFLD patients with fibrosis (*n* = 21), NAFLD patients without fibrosis (*n* = 33), and healthy controls (*n* = 20). *P* = 0.903 for control versus NAFLD without fibrosis, *P* = 0.028 for control versus NAFLD with fibrosis, and *P* = 0.032 for NAFLD with fibrosis versus NAFLD without fibrosis. Pentraxin 3 levels were less than 2 ng/mL (cutoff) in both NAFLD patients without fibrosis and control subjects.

Patients with NAFLD were divided into two subgroups according to presence/absence of fibrosis. Comparisons of demographic, biochemical, and vascular characteristics among the three study groups were summarized in Table 2. The distribution of age, gender, and smoking were similar among the three groups (*P* > 0.05). SBP and LDL-c and HDL-c were similar among the groups. Compared to control subjects, NAFLD patients, regardless of fibrosis status, had higher BMI, WC, DBP, FPG, and hs-CRP levels but no significant difference in these parameters was found between the NAFLD subgroups. NAFLD patients with fibrosis had higher 2 h OGTT, insulin and HOMA-IR levels compared to controls, but no significant difference in these parameters existed for other comparisons (Table 2). PTX3 levels in NAFLD patients with fibrosis were higher than both NAFLD patients without fibrosis and controls (*P* = 0.032 and *P* = 0.028, resp.), but there was no significant difference between controls and NAFLD patients without fibrosis in terms of PTX3 levels (*P* = 0.903) (Figure 1).

In assessment of subclinical atherosclerosis markers, cf-PWV levels in NAFLD patients with fibrosis were higher than the control subjects (*P* = 0.010). However, cf-PWV levels in NAFLD patients without fibrosis were similar compared to NAFLD patients with fibrosis and control subjects (*P* = 0.495 and *P* = 0.063, resp.). FMD levels in both NAFLD patients with and without fibrosis were lower than the controls (*P* = 0.008 for both comparisons), but no significant difference in FMD levels existed between the NAFLD subgroups (*P* = 0.976). NAFLD patients with fibrosis had higher CIMT levels compared to NAFLD patients without fibrosis (*P* = 0.023), but no significant difference in CIMT levels occurred in other comparisons (*P* > 0.05) (Table 2).

To evaluate whether elevated PTX3 or hs-CRP levels could predict fibrosis in NAFLD, a logistic regression model for multivariate analysis was carried out on the basis of clinical significance criteria. In model 1, PTX3, ALT, and age were independently associated with presence of fibrosis in NAFLD after adjustment for age, smoking status, systolic and diastolic blood pressure, uric acid, AST, ALT, ALP, GGT, ferritin, and hs-CRP (*P* = 0.032, *P* = 0.002, and *P* = 0.01, resp.). Additional analyses were conducted adjusting for components of metabolic syndrome to test for a true independent effect of fibrosis on increased inflammation (model 2). PTX3 levels remained significant with approximately 1.5-fold difference (Table 3).

**Figure 2:**
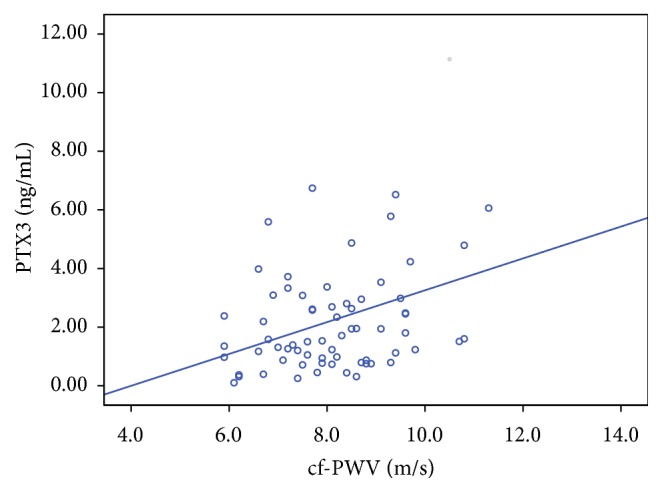
Correlation between pentraxin 3 and cf-PWV (arterial stiffness) in the study population (*r* = 0.359; *P* = 0.003).

In correlation analysis, PTX3 levels were strongly and positively correlated with cf-PWV (*r* = 0.359; *P* = 0.003) (Figure 2), whereas no significant correlation was found with other atherosclerosis markers, CIMT and FMD. In addition, PTX3 levels did not correlate with levels of the short pentraxin CRP, BMI, FPG, LDL, and insulin levels. There was a weak but statistically significant negative correlation between PTX3 and HDL-c (*r* = −0.266; *P* = 0.027).

## 4. Discussion

In the current study, we have demonstrated that PTX3 levels in NAFLD patients with fibrosis were higher than NAFLD patients without fibrosis and healthy subjects, independent of metabolic syndrome components. We have also demonstrated that PTX3 levels were strongly correlated with arterial stiffness in patients with NAFLD. To the best our knowledge, this is the first study that evaluated the association between plasma PTX3 concentrations and vascular parameters in patients with NAFLD. Thus, it suggests that PTX3 may become a useful marker to understand the development of fibrosis in patients with NAFLD and a biochemical indicator of increased arterial stiffness in subclinical atherosclerosis. In addition, the results of the present study showed that NAFLD patients with fibrosis carry a high risk for increased arterial stiffness and endothelial dysfunction as well as elevated CIMT levels. These data imply that the presence of fibrosis in patients with NAFLD may be a risk factor for atherosclerosis.

While the pathophysiology of NAFLD remains incompletely understood, increased hepatic insulin resistance and subclinical inflammation are believed to play an integral role in the pathogenesis and progression of NAFLD [20]. In fact, the distinction between NASH and SS is of great importance because NASH and the concomitant presence of fibrosis are considered to be a disease that can lead to cirrhosis, whereas SS normally does not progress [21]. A specific biomarker that can detect presence of fibrosis would be of great clinical usefulness. Several clinical studies have reported that hs-CRP may be an independent indicator for progression of SS to NASH [22]. Nonetheless, the relevance of CRP as a predictor of fibrosis in NAFLD has not been well established yet. PTX3 shares some similarities with short pentraxins, but differs in terms of structural domain, gene organization, cellular and tissue sources, inducing stimuli, and recognized ligands [23]. To date, PTX3 levels were usually investigated by the research studies in assessment of CVD [24, 25]. Diagnostic and prognostic value of plasma PTX3 level in patients with NAFLD have been evaluated in three previous studies with controversial results [13–15]. In the present study, we found that PTX3 levels were higher in NAFLD patients with fibrosis compared to NAFLD patients without fibrosis and control subjects. PTX3 levels were also less than 2 ng/mL in both NAFLD without fibrosis and control subjects. On the other hand, we showed that hs-CRP levels were elevated in patients with NAFLD, regardless of fibrosis status, compared to healthy subjects, but no significant difference between NAFLD subgroups was existing. We think that hs-CRP may be a marker of steatosis, but not of fibrosis in NAFLD. Our study supports the results of Zimmermann et al., who established that elevated hs-CRP was associated with steatosis in NAFLD [26]. Therefore, these data suggest that increased plasma PTX3 concentration may be a more valuable indicator for prediction of fibrosis than hs-CRP in patients with NAFLD. Since NASH is one of the major causes of liver-related mortality and morbidity, the results of the current study provide important information, suggesting that plasma PTX3 level is one of the candidates of the target for therapeutic interventions and monitoring to improve the clinical outcome in the high risk population.

A growing body of evidence suggest that CVD is the most important cause of death in patients with NAFLD [27]. We and others have reported that NAFLD is associated with an increased risk of impaired FMD and increased CIMT and arterial stiffness, independently of traditional risk factors and metabolic syndrome [11, 28]. The association between severity of NAFLD (fibrosis staging and necroinflammation grading) and atherosclerosis is not fully elucidated. Moreover, there is no information regarding the role of PTX3 in assessment of cardiovascular risk in patients with NAFLD. In the present study, NAFLD patients with fibrosis had increased arterial stiffness and impaired endothelial function compared to the healthy controls. Also, we have shown that presence of fibrosis is a risk factor for increased CIMT levels in patients with NAFLD. In fact, our findings are consistent with results of previous studies [28, 29]. Furthermore, we investigated a novel association between PTX3 and vascular parameters in patients with NAFLD. In fact, the previous studies have investigated the relationship between arterial stiffness and PTX3 in various conditions. While some of them found that PTX3 is negatively correlated with arterial stiffness in obese individuals and adults during an 8-week aerobic exercise intervention [30, 31]; the remaining study showed that PTX3 levels were higher in individuals with increased arterial stiffness [32]. The present study has shown that there is a statistically significant correlation between PTX3 and arterial stiffness. We think that inflammation can play a central role in NAFLD and arterial stiffness. Recent studies have shown that inflammation has been associated through either causal or associative relationships with increased arterial stiffness [33, 34]. It could be speculated that the association between PTX3 and cf-PWV may reflect a state of atherosclerosis. However, plasma PTX3 levels were not associated with other surrogate markers of atherosclerosis, including CIMT and FMD. To explain this discrepancy, we postulate that increased CIMT and impaired FMD levels may not always reflect inflammatory changes in the artery wall, whereas arterial stiffness is closely associated with subclinical inflammatory process [35].

This study has several strengths and limitations. Strengths include the presence of liver biopsy for diagnosis of NAFLD and availability of the measurements of cf-PWV, FMD, and CIMT to detect subclinical atherosclerosis. The first limitation is that the number of participants with fibrosis was quite small. Therefore, we could not establish the sensitivity and specificity analysis for the diagnostic performance of PTX3 levels in assessment of fibrosis. Secondly, because of the cross-sectional nature of this study, the associations do not necessarily indicate causality. Lastly, we used fasting insulin and HOMA-IR to evaluate insulin resistance rather than the gold standard clamp technique. Considering a linear correlation existing between HOMA-IR and measurement of insulin sensitivity using the glucose clamp technique, this commonly used index is routinely used to provide a noninvasive assessment of insulin sensitivity.

In summary, elevated plasma PTX3 levels are associated with the presence of fibrosis in patients with NAFLD, independently of metabolic syndrome components and fibrosis predictors. NAFLD patients with fibrosis have increased risk of atherosclerosis when compared to NAFLD patients without fibrosis and healthy controls. In addition, this study demonstrated that for the first time there is a close association between elevated PTX3 levels and increased arterial stiffness in patients with NAFLD.

## Figures and Tables

**Table 1:** Baseline characteristics of study population.

Variables	NAFLD (*n* = 54)	Control (*n* = 20)	*P*
Age (year)	33.8 ± 5.9	30.8 ± 5.1	0.054
Smoking (%)	37.7	22.2	0.265
BMI (kg/m^2^)	29.4 ± 3.2	23.8 ± 2.7	<0.001
WC (cm)	101.1 ± 8.1	89 ± 10.1	<0.001
HC (cm)	106.3 ± 6.8	99.8 ± 5.4	<0.001
SBP (mmHg)	129.4 ± 9.6	117.3 ± 8.4	0.023
DBP (mmHg)	80.3 ± 7.9	71.1 ± 5.8	<0.001
AST (U/L)^*∗*^	61.9 ± 48.2	22.1 ± 4.9	<0.001
ALT (U/L)	107.2 ± 55.5	22.7 ± 8.1	<0.001
GGT (U/L)	70.9 ± 42.8	20.7 ± 7.9	<0.001
FPG (mg/dL)	97.8 ± 10.3	88.2 ± 6.6	0.001
2 h OGTT (mg/dL)	106.3 ± 26.6	91.1 ± 13.6	0.034
Creatinine (mg/dL)	1 ± 0.1	1 ± 0.1	0.332
Uric acid (md/dL)	6.9 ± 1.2	5.4 ± 0.9	<0.001
LDL-C (mg/dL)	129.4 ± 30.6	106.8 ± 33.3	0.011
HDL-C (mg/dL)^*∗*^	42.4 ± 7.8	43.6 ± 10.1	0.795
TG (mg/dL)	183.4 ± 103.7	142.6 ± 103.3	0.156
Insulin (mU/mL)	17.4 ± 10	10.7 ± 5.5	0.014
HOMA-IR	4.3 ± 2.8	2.3 ± 1.1	0.007
Ferritin (ng/mL)^*∗*^	166.1 ± 159.8	79.4 ± 57.3	0.003
hs-CRP (mg/dL)^*∗*^	4.1 ± 3.7	1.7 ± 1.1	0.006
PTX3 (ng/mL)	2.4 ± 1.9	1.7 ± 1.4	0.156
cf-PWV (m/s)	8.3 ± 1.2	7.3 ± 1.2	0.006
CIMT (mm)	0.451 ± 0.07	0.441 ± 0.07	0.653
FMD (%)^*∗*^	11.2 ± 6.9	20.1 ± 11.9	0.014

^*∗*^Mann-Whitney *U* test was used for nonparametric tests.

NAFLD: nonalcoholic fatty liver disease, WC: waist circumference, HC: hip circumference, BMI: body mass index, SBP: systolic blood pressure, DBP: diastolic blood pressure, AST: aspartate aminotransferase, ALT: alanine aminotransferase, GGT: *γ*-glutamyltransferase, FPG: fasting plasma glucose, OGTT: oral glucose tolerance testing, LDL-C: low density lipoprotein cholesterol, HDL-C: high density lipoprotein cholesterol, TG: triglyceride, HOMA-IR: homeostasis model assessment of insulin resistance, hs-CRP: high sensitive c reactive protein, PTX3: pentraxin 3, cf-PWV: carotid femoral pulse wave velocity, CIMT: carotid intima media thickness, and FMD: flow mediated dilatation.

**Table 2:** The assessment of vascular and metabolic parameters according to the presence of fibrosis.

Variables	NAFLD with fibrosis (*n* = 21)	NAFLD without fibrosis (*n* = 33)	Controls (*n* = 20)	*P*
Age (year)	34.3 ± 6.5	33.5 ± 5.5	30.8 ± 5.1	0.138
Smoking (%)	35	39.4	22.2	0.460
BMI (kg/m^2^)	29.9 ± 2.8	29.1 ± 3.2	23.8 ± 2.7	<0.001
WC (cm)	108 ± 5.6	105.4 ± 7.3	89 ± 10.1	<0.001
SBP (mmHg)	131.3 ± 9.9	128.3 ± 24.4	117.3 ± 8.4	0.065
DBP (mmHg)	82 ± 5.9	79.3 ± 8.9	71.1 ± 5.8	<0.001
FPG (mg/dL)	100.1 ± 10.3	96.3 ± 10.3	88.2 ± 6.6	0.001
2 h OGTT (mg/dL)	115.7 ± 30.3	100.7 ± 22.7	91.1 ± 13.6	0.013
LDL-C (mg/dL)	129.2 ± 21.1	129.6 ± 35.4	106.8 ± 33.3	0.059
HDL-C (mg/dL)	38.6 ± 9.3	44.6 ± 9.2	43.6 ± 10.1	0.068
Insulin (mU/mL)	19.1 ± 8.8	16.3 ± 10.6	10.7 ± 5.5	0.028
HOMA-IR	4.8 ± 2.4	4 ± 3	2.3 ± 1.1	0.015
hs-CRP (mg/dL)	3.7 ± 2.2	4.4 ± 4.4	1.7 ± 1.1	0.024
PTX3 (ng/mL)	3.2 ± 2.6	1.9 ± 1.2	1.7 ± 1.4	0.016
cf-PWV (m/s)	8.6 ± 1.3	8.2 ± 1.1	7.3 ± 1.2	0.012
CIMT (mm)	0.485 ± 0.06	0.427 ± 0.08	0.441 ± 0.07	0.028
FMD (%)	11 ± 8	11.5 ± 6.3	20.1 ± 11.9	0.005

NAFLD: nonalcoholic fatty liver disease, BMI: body mass index, WC: waist circumference, SBP: systolic blood pressure, DBP: diastolic blood pressure, hs-CRP: high sensitive c reactive protein, FPG: fasting plasma glucose, OGTT: oral glucose tolerance testing, LDL-C: low density lipoprotein cholesterol, HDL-C: high density lipoprotein cholesterol, TG: triglyceride, HOMA-IR: homeostasis model assessment of insulin resistance, PTX3: pentraxin 3, cf-PWV: carotid femoral pulse wave velocity, CIMT: carotid intima media thickness, and FMD: flow mediated dilatation.

**Table 3:** Logistic regression analysis of predictors associated with fibrosis in NAFLD.

Variables	Model 1				Model 2			
	OR	95% CI		*P*	OR	95% CI		*P*
		Lower	Upper			Lower	Upper	
Age	1.391	1.084	1.785	0.010				
ALT	1.042	1.015	1.070	0.002				
PTX3	1.712	1.047	2.800	0.032	1.545	1.040	2.295	0.031
WC					1.169	1.043	1.311	0.007
Constant	<0.001			0.002	<0.001			0.003

Model 1: adjusted for age, smoking status, systolic and diastolic blood pressure, uric acid, AST, ALT, ALP, GGT, ferritin, and hs-CRP.

Model 2: adjusted for BMI, waist and hip circumference, glucose, 2 h OGGT, insulin, HOMA-IR, and lipids. Bolded items are significant (*P* < 0.05).
